# Supplementary material for: Sterol regulatory element binding protein 1 couples mechanical cues and lipid metabolism
Source: Nat Commun. 2019 Mar 22;10:1326. doi: 10.1038/s41467-019-09152-7 (PMC6430766; doi:10.1038/s41467-019-09152-7)
Supplement: Supplementary file 1 — Supplementary Information [file 41467_2019_9152_MOESM1_ESM.pdf]

Supplementary Information

Sterol Regulatory Element Binding Protein 1 couples mechanical cues and lipid metabolism

Bertolio et al.

Supplementary figures

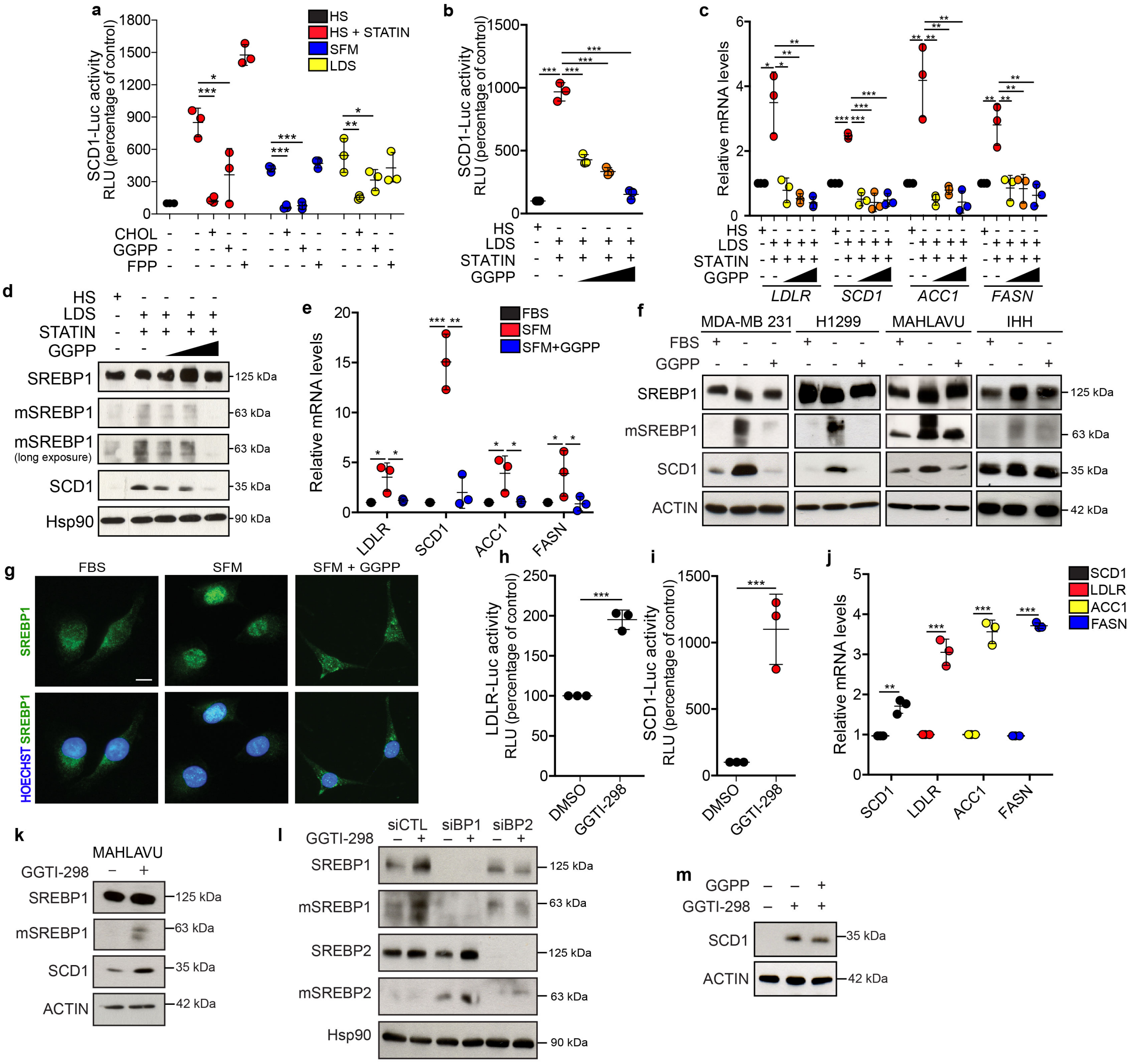

Supplementary Fig. 1 | a, SCD1-Luc assay in MCF-10A cells treated with cholesterol (CHOL), geranylgeranyl (GGPP) or farnesyl pyrophosphate (FPP). 5% horse serum (HS) medium (control) was supplemented with 10 µM cerivastatin (STATIN), replaced with serum-free (SFM), or 2% lipid serum (lipid-depleted serum, LDS) medium. **b**-**d**, SCD1-Luc assay (**b**), *LDLR*, *SCD1, ACC1* and *FASN* RT-qPCR (**c**) SREBP1 and SCD1 western blot (**d**) in MCF-10A cells. 5% HS medium (control) was replaced with 2% LDS medium supplemented with 1 µM cerivastatin (STATIN) and 20, 40, 100 µM GGPP. **e**, *LDLR, SCD1, ACC1* and *FASN* RT-qPCR in IHH cells. 5% HS medium (control) was replaced with SFM or SFM supplemented with GGPP. **f**, SREBP1 and SCD1 western blot in MDA-MB 231,

H1299, Mahlavu and IHH cells cultured in 10% foetal bovine serum (FBS) medium, SFM, or SFM supplemented with GGPP. **g**, SREBP1 Immunofluorescence (green) in MDA-MB 231 cells cultured in 10% FBS medium, SFM, or SFM supplemented with GGPP. Nuclei were stained with HOECHST (in blue). Scale bar 15 µm. **h** LDLR-Luc assay in IHH cells treated with DMSO (control) or geranylgeranyl transferase I inhibitor (GGTI-298). **i**, SCD1-Luc assay in MCF-10A cells treated with DMSO (control) or 5µM GGTI-298. **j,** *LDLR*, *SCD1*, *ACC1* and *FASN* RT-qPCR in IHH cells treated with DMSO (control) or 5µM GGTI-298. **k**, Western blot analysis of Mahlavu cells treated with DMSO (control) or 5µM GGTI-298. **l**, Western blot analysis of MCF-10A cells transfected with control (siCTL), SREBP1 (siBP1) or SREBP2 (siBP2) siRNA and treated with GGTI-298. **m**, Western blot analysis of MCF-10A cells treated with DMSO, GGTI-298 or GGTI-298 and GGPP.

Graph bars represent mean ± s.d. of n=3 biological replicates. Treatments lasted 24 hours Values in **a**, **b**, **h** are expressed as Relative Luminometer Units (RLU). RT-qPCR values are expressed as mRNA levels relative to control. P value: *P < 0.05, **P < 0.01, ***P < 0.001 by two-tailed Student's t –test for all the analyses. Blots are representative of n=3 biological replicates. For western blots, mSREBP indicates mature protein, Actin and Hsp90 were loading controls.

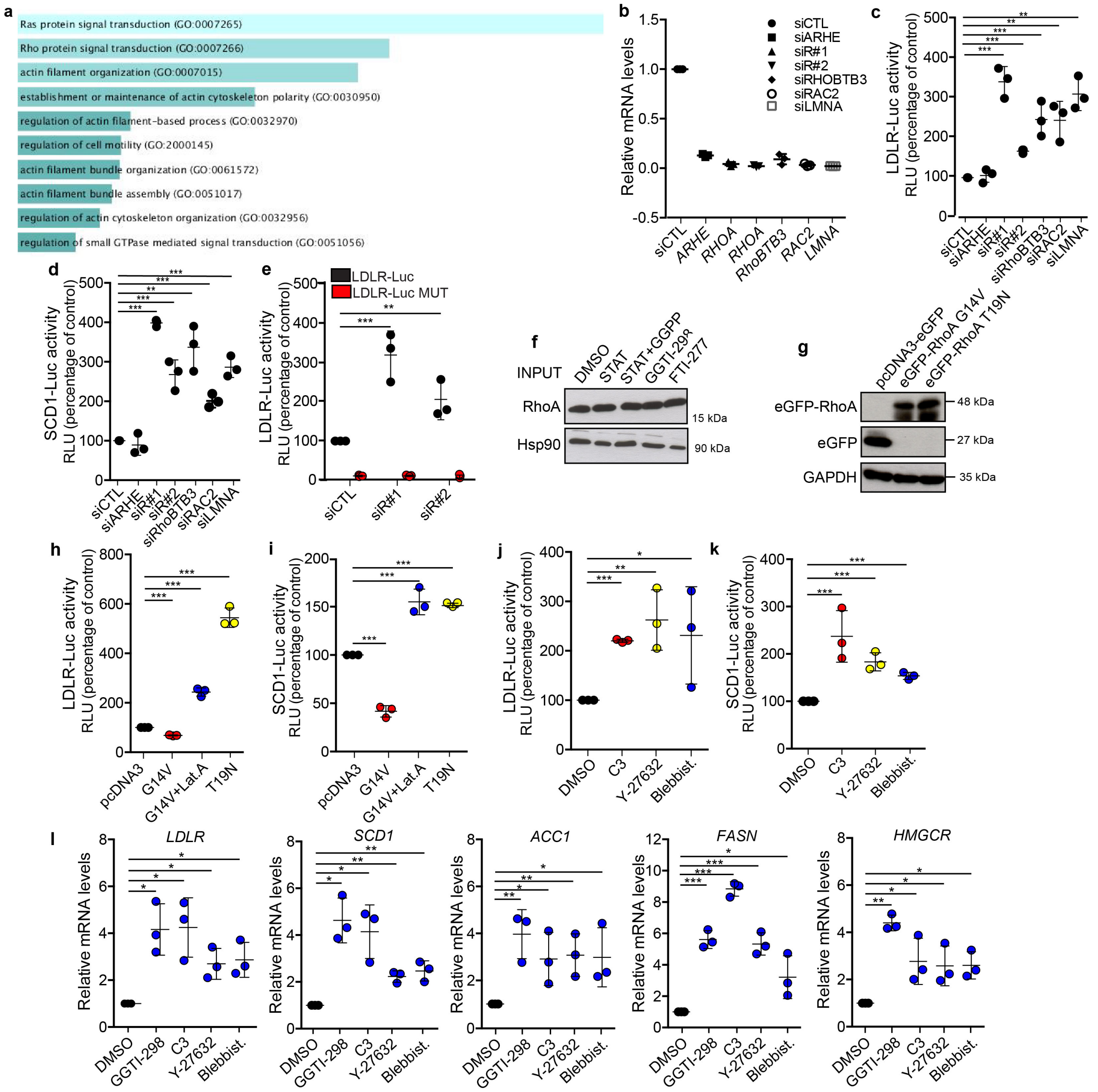

Supplementary Fig. 2 | a, Gene ontology Biological Process categories that are significantly enriched in geranylgeranylated proteins that negatively regulate SREBP. **b**, *RhoE/AHRE, RhoA, RhoBTB3, Rac2 and Lamin A* RT-qPCR in MDA-MB-231, 48h after transfection with control siRNA (siCTL) or siRNAs targeting *RhoE (siARHE), RhoA (siR#1 and siR#2), RhoBTB3 (siRHOBTB3), Rac2 (siRAC2) and Lamin A (siLMNA).* **c**, LDLR-Luc assay in MDA-MB 231 cells transfected with siCTL siARHE, siRhoA#1, siRhoA#2, siRHOBTB3, siRAC2 or siLMNA. **d**, SCD1-Luc assay in MDA-MB 231 cells transfected with either siCTL siARHE, siRhoA#1, siRhoA#2, siRHOBTB3, siRAC2 or siLMNA. **e,** LDLR-Luc assay in IHH cells transfected with siCTL, siRhoA#1, or siRhoA#2, Cells transfected with the mutated construct LDLR-Luc MUT underwent the same treatments. **f,** Western blot analysis of

MCF-10A cells treated DMSO (as control), 1 µM cerivastatin (STAT), 1 µM cerivastatin and 20 µM GGPP (STAT+GGPP), 5 µM GGTI-298 or 5 µM FTI-277. **g,** Western blot analysis of MCF-10A cells transfected with pcDNA3-eGFP, pcDNA3-eGFP-RhoA G14V and pcDNA3-eGFP-RhoA T19N. **h,** LDLR-Luc assay in IHH cells (**h**), SCD1-Luc assay in MCF-10A cells (**i**) 12 hours after transfection with pcDNA3-GFP control plasmid, pcDNA3-GFP-RhoA G14V construct with a 6 hours DMSO treatment, pcDNA3-GFP-RhoA G14V construct with a 6 hours Latrunculin A (G14V + Lat.A) treatment, or pcDNA3-GFP-RhoA T19N construct. **j, k, l**, LDLR-Luc assay in IHH cells (**j**), SCD1-Luc assay (**k**), *LDLR*, *SCD1*, *ACC1*, *FASN and HMGCR* RT-qPCR (**l**) in MCF-10A cells treated with DMSO (control), GGTI-298, C3, Y-27632 or Blebbistatin (Blebbist.) for 24 hours.

Graph bars represent mean ± s.d. of n=3 biological replicates. Values in **c**-**e**, **h**-**k** are expressed as Relative Luminometer Units (RLU). RT-qPCR values are expressed as mRNA levels relative to control. Blots are representative of n=3 biological replicates. P value: *P < 0.05, **P < 0.01, ***P < 0.001 by two-tailed Student's t-test for all analyses. For western blots, GAPDH and Hsp90 were loading controls.

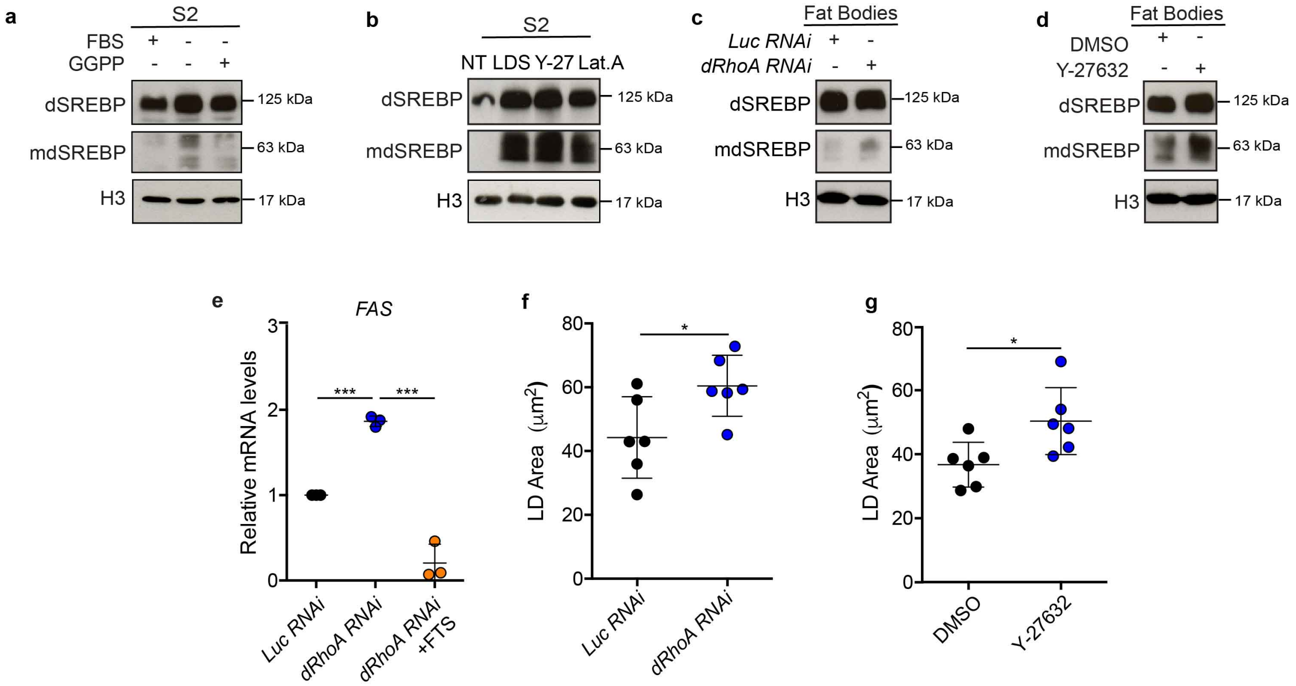

Supplementary Fig. 3 | a, Western blot analysis of S2 *Drosophila* cells treated as follows: cell culture medium containing 10% foetal bovine serum (FBS) was replaced with serum-free medium or serum-free medium supplemented with GGPP. **b**, Western blot analysis of S2 *Drosophila* cells treated as follows: cell culture medium containing 10% FBS (non-treated, NT) was either replaced with medium containing 2% lipid serum (lipid-depleted serum, LDS), or supplemented with Y-27632 (Y27) or Latrunculin A (Lat.A). **c**, Western blot analysis of *Drosophila* larval fat bodies expressing *Luciferase RNAi* (Luc) or *dRhoA RNAi*. **d**, Western blot analysis of wild type *Drosophila* larval fat bodies treated with either DMSO or Y-27632 for 16 hours. **e**, RT-qPCR quantification of the expression of the *Fatty Acid Synthase* (*FAS*) dSREBP target gene in *Drosophila* larval fat bodies expressing *Luc RNAi* (as control, in black), *dRhoA RNAi* (in blue), or *dRhoA RNAi* and treated with fatostatin for 16 hours (FTS, in orange). Values are expressed as mRNA levels relative to control. Bars represent mean value ± s.d. of n=3 biological replicates. *Rp49* was used as reference gene. **f**, Quantification of lipid droplet size in *Drosophila* larval fat bodies expressing *Luc RNAi* (as control, in black), *dRhoA RNAi* (in blue), or *dRhoA RNAi* and treated with fatostatin for 16 hours (FTS, in orange). Representative images are shown in Figure 2h. **g**, Quantification of lipid droplet size in wild type *Drosophila* larval fat bodies treated with either DMSO (in black) or Y-27632 (in blue). Representative images are shown in Fig. 2h.

Graph bars represent mean value ± s.d. of n=3 biological replicates (**e**) or n=6 individuals (**f** and **g**). For all western blots, H3 was used as loading control. Blots are representative of n=3 biological

replicates. For western blots, mSREBP indicates mature protein. P value: *P < 0.05, ***P < 0.001 by two-tailed Student's t-test for all analyses.

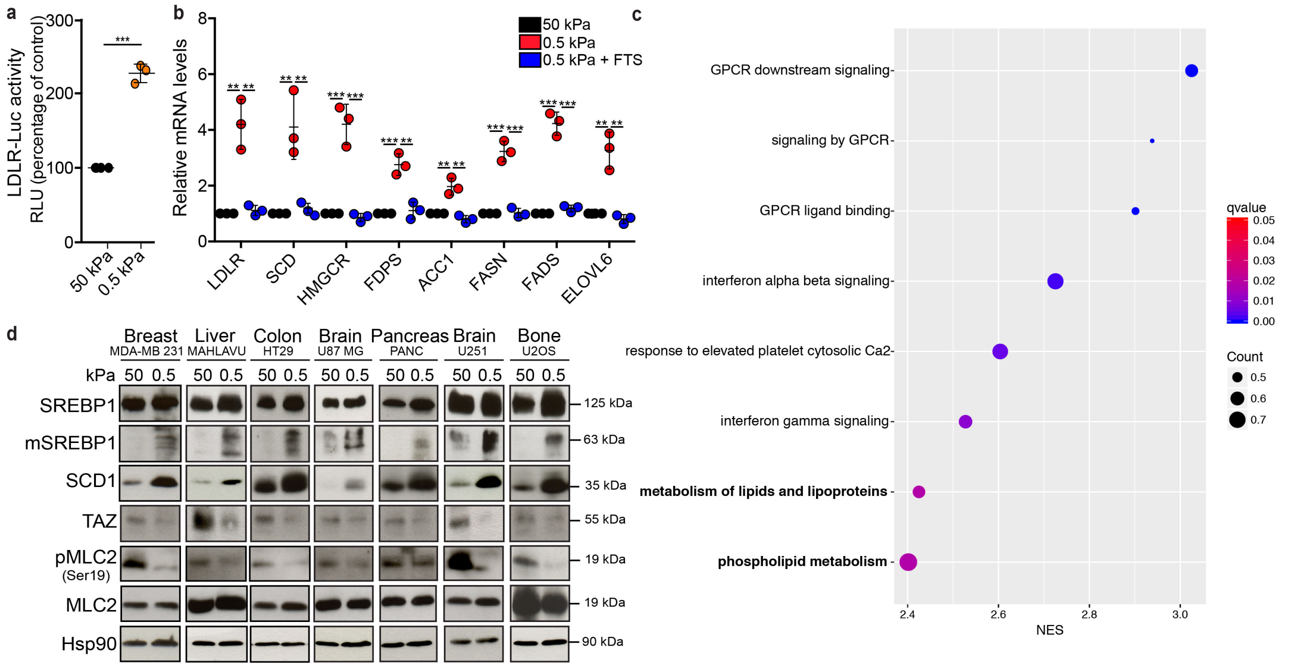

Supplementary Fig. 4 | a, Low density lipoprotein promoter-luciferase (LDLR-Luc) reporter assay in IHH cells cultured on either stiff (50 kPa elastic modulus, as control) or soft (0.5 kPa elastic modulus) fibronectin-coated hydrogel matrix for 24 hours. Graphs bars represent mean value ± s.d. of n=3 biological replicates. P value: *P < 0.001 by two-tailed Student's t-test for all analyses. **b**, RT-qPCR quantification of the expression of the indicated genes in IHH cells cultured on either stiff (50 kPa elastic modulus, as control) or soft (0.5 kPa elastic modulus) fibronectin-coated hydrogel matrix, or soft fibronectin-coated hydrogel matrix with fatostatin treatment (FTS), for 24 hours. Graphs bars represent mean value ± s.d. of n=3 biological replicates. P value: **P < 0.01, ***P < 0.001 by two-tailed Student's t-test for all analyses. **c**, Reactome gene sets that are significantly enriched (FDR q value < 0.05) in MDA-MB-231 cells cultured on a soft substrate. Dot size represents the fraction of genes contributing to the enrichment score in each gene set (count); the positive normalized enrichment score (NES) indicates the degree to which Reactome gene sets are overrepresented in cells grown on the soft (as compared to cells grown on the stiff) substrate. Gene expression data were obtained from n=4 biological replicates for each condition (GSE93529). **d**, Western blot analysis of cell lines originated from the indicated tissues, cultured on either stiff (50 kPa elastic modulus) or soft (0.5 kPa elastic modulus) fibronectin-coated hydrogel matrix for 24 hours. Hsp90 was used as loading control. Blots are representative of n=3 biological replicates. mSREBP indicates mature protein.

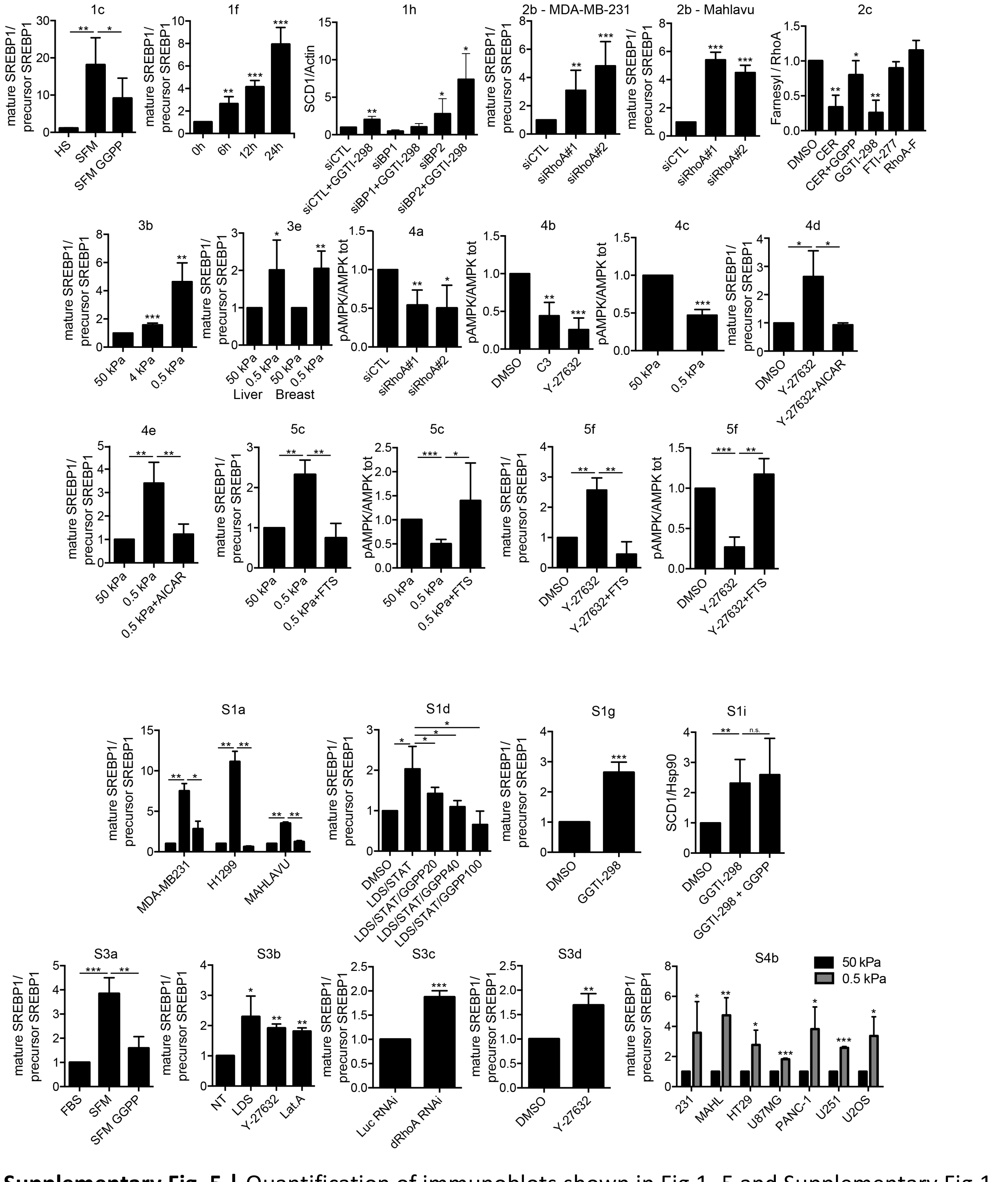

Supplementary Fig. 5 | Quantification of immunoblots shown in Fig.1 -5 and Supplementary Fig.1-4. *P ≤ 0.05, **P < 0.01, ***P < 0.001 by two-tailed Student's t -test.

Figure 1

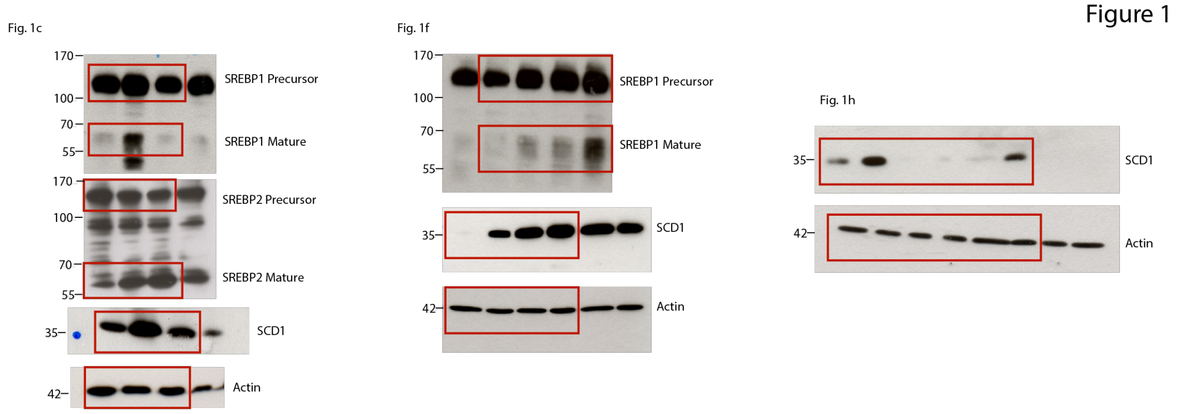

Figure 2

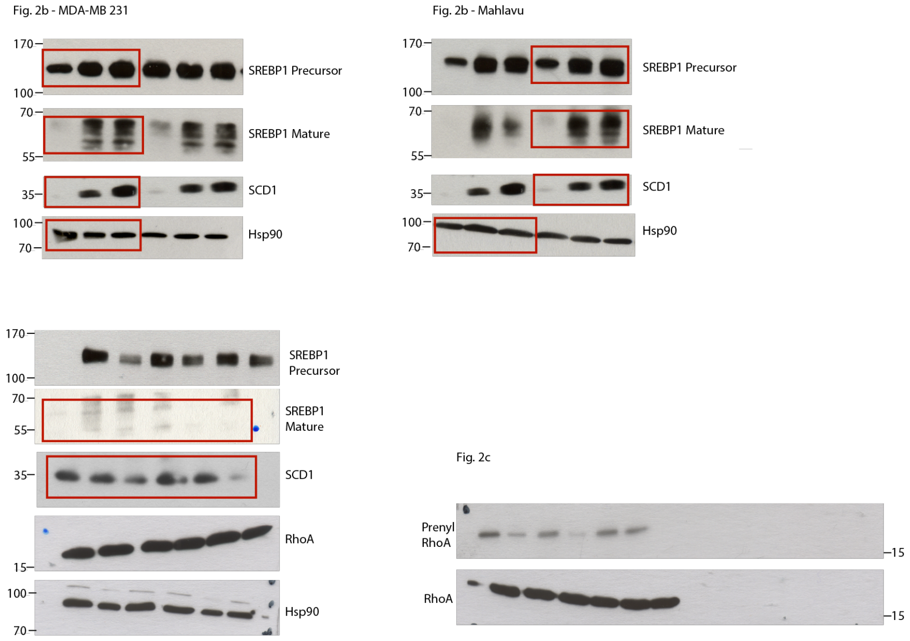

Figure 3

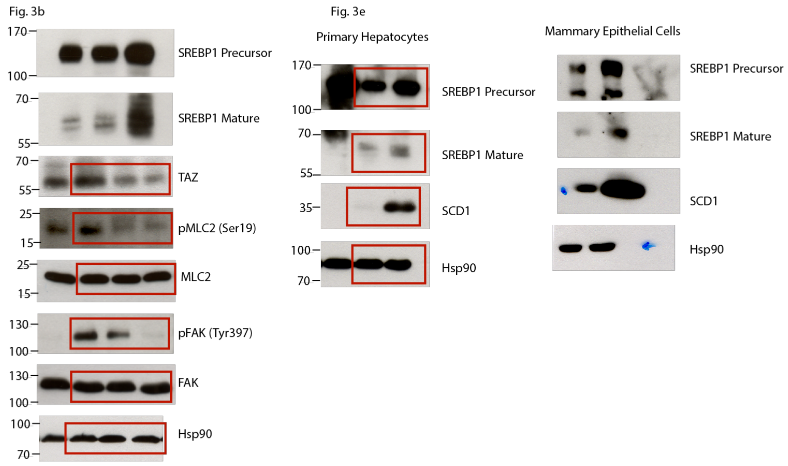

Supplementary Fig. 6 | Uncropped blots from Fig.1, 2, 3, 4, 5 and Supplementary Fig. 1, 2, 3 and 4.

Figure 4

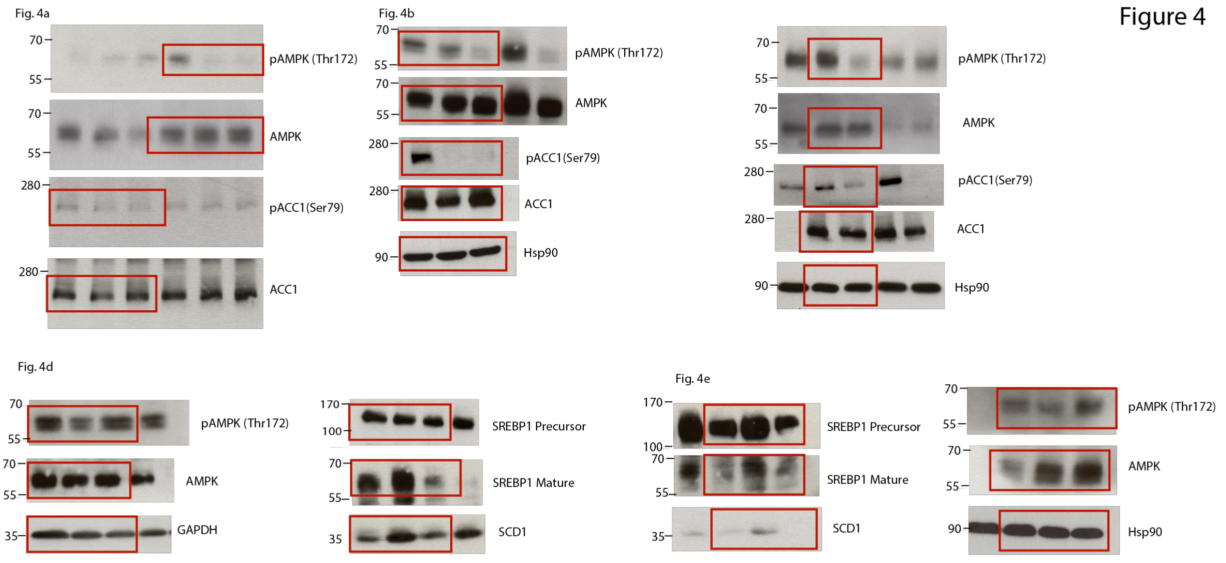

Figure 5

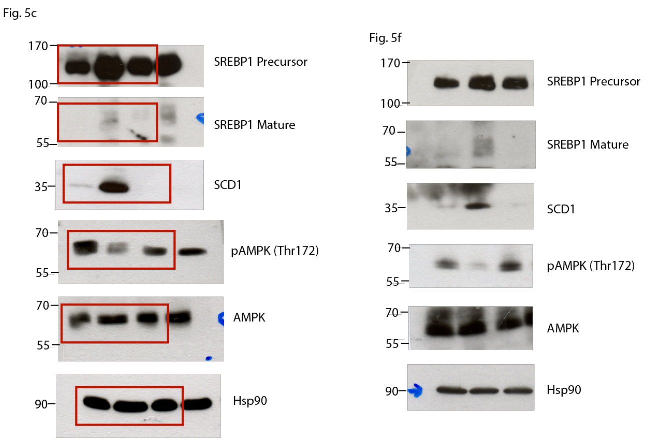

Supp. Fig.1

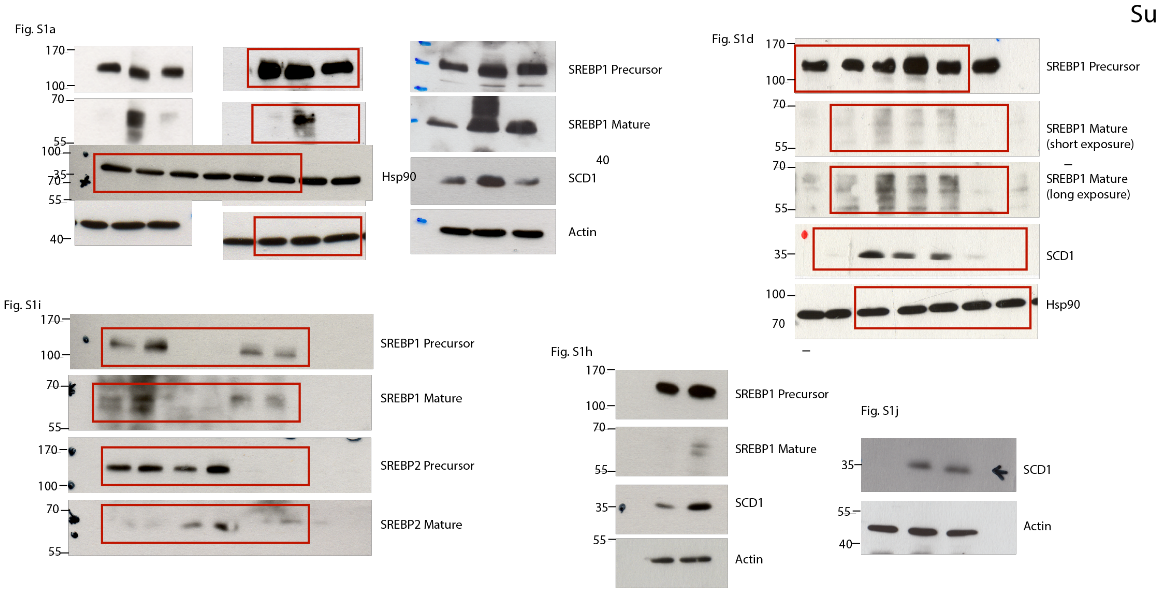

Supplementary Fig. 6 | Continued.

Supp. Fig.2

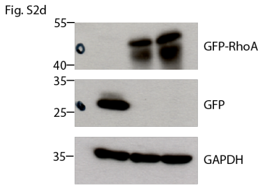

Supp. Fig.3

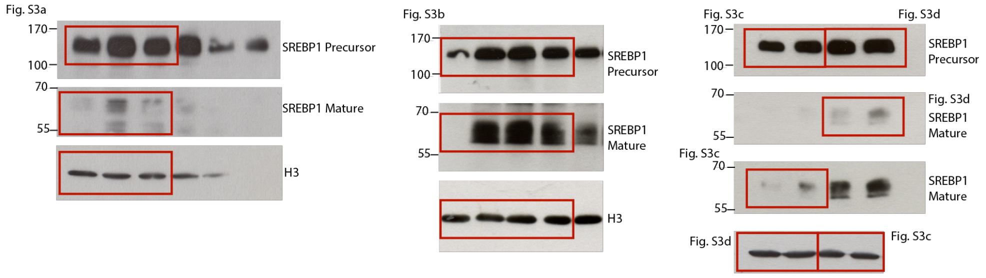

Supp. Fig.4

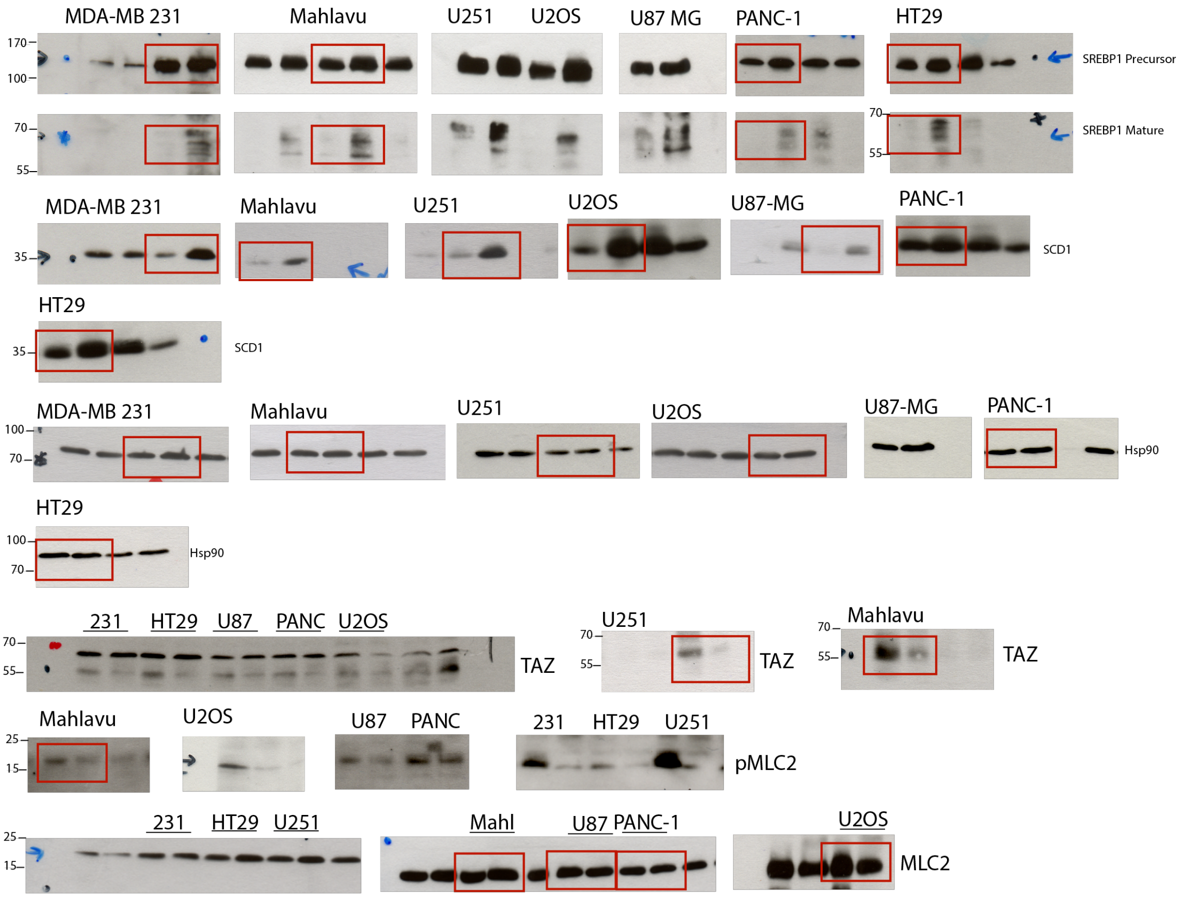

Supplementary Fig. 6 | Continued.

Supplementary tables

Supplementary Table 1 | List of siRNAs sequences used

Human SREBP1	siSREBP-1	AUCUCUGAAGGAUCUGGUG
Human SREBP2	siSREBP-2	GCCCUCUAUUGGAUGAUGC
Human RhoA	siRhoA#1	AUGGAAAGCAGGUAGAGUU
Human RhoA	siRhoA#2	GAAAGACAUGCUUGCUCAU
Human ARHE	siARHE	UAGUAGAGCUCUCCAAUCACA
Human RhoBTB3	siRhoBTB3	AGGAAGAAGUUGAAAGAUUUU
Human RAC2	siRAC2	CCUCUUUUGGAACAACAUA
Human LMNA	siLMNA	GGAUGAGGAUGGAGAUGAC

Supplementary table 2 | List of primers used for qRT-PCR

human *LDLR*	Fw	AAGCCATTCACTTCCCCAATC
	Rv	GCCTCACCGTGCATGTTTTA
human *SCD1*	Fw	CACTTGGGAGCCCTGTATGG
	Rv	TGAGCTCCTGCTGTTATGCC
human *HMGCR*	Fw	GGACCCCTTTGCTTAGATGAAA
	Rv	CCACCAAGACCTATTGCTCTG
human *FDPS*	Fw	CTTCCTATAGCTGCAGCCATGTAC
	Rv	GCATTGGCGTGCTCCTTCT
human *ACC1*	Fw	CATATTGAGGATGACAGGCTGG
	Rv	CTCATAGTTGACCTGCTTTCTG
human *FASN*	Fw	CATCCAGATAGGCCTCATAGA
	Rv	CTCCATGAAGTAGGAGTGGAA
human *FADS1*	Fw	GCTACTTCACCTGGGACGAG
	Rv	GTGGAAGGCCACAAAGGGAT
human *ELOVL6*	Fw	CTCGAAATCAAGCGCTTTACAGA
	Rv	AGGCAGCATACAGAGCAGAAA
human *RhoA*	Fw	ATTCGTTGCCTGAGCAATGG
	Rv	TGTGTCCCACAAAGCCAACT
human ARHE	Fw	GCCAGCCAGAAATTATCCAGC
	Rv	CTTGGCGAAGACATGGAGC
human RHOBTB3	Fw	ACTCCACAGCCTTGATGACTT
	Rv	AAGCACCTGGTTGTTCAAGTT
human RAC2	Fw	CGCCAAGTGGTTCCCAGAAG
	Rv	AGCTGAGCACTCCAGGTATTT
human *H3*	Fw	GTGAAGAAACCTCATCGTTACAGGCCTGGT
	Rv	CTGCAAAGCACCAATAGCTGCACTCTGGAA
mouse *Pparg*	Fw	AAGAGCTGACCCAATGGTTG
	Rv	ACCCTTGCATCCTTCACAAG
mouse *AdipoQ*	Fw	GACAAGGCCGTTCTCTTCAC
	Rv	CAGACTTGGTCTCCCACCTC
mouse *Cebpa*	Fw	GAACAGCAACGAGTACCGGGTA
	Rv	GCCATGGCCTTGACCAAGGAG
mouse *Fabp4*	Fw	AAGTGGGAGTGGGCTTTGC
	Rv	CCGGATGGTGACCAAATCC
mouse *Gapdh*	Fw	ATCCTGCACCACCAACTGCT
	Rv	GGGCCATCCACAGTCTTCTG
Drosophila FAS	Fw	CCCCAGGAGGTGAACTCTATCA
	Rv	GACTTGACCGATCCGATCAAC
Drosophila Rp49	Fw	ATCGGTTACGGATCGAACAA

	Rv	GACAATCACCTTGCGCTTCT
Drosophila RhoA	Fw	GTGGATGGCAAACAGGTGGAGC
	Rv	GCGAATCGGGTGAATCCACTGAG

Supplementary table 3 | List of proteins that negatively regulate LDLR-Luc upon siRNA silencing

Protein Name	Gene ID	Fluc/Rluc, fold over siCTL
ARHE	390	9,446
RHOA	387	8,590
RHOBTB3	22836	8,568
RAC2	5880	6,500
LMNA	4000	5,802
KIAA1164	54629	5,448
RHOB	388	5,253
FLJ23878	200172	4,339
GNG13	51764	4,264
RAP1A	5906	4,136
UBL3	5412	4,083
FLJ11280	55793	4,013
LMNB2	84823	3,898
RHOJ	57381	3,551
GBP5	115362	3,345
MRAS	22808	3,126
RHOG	391	3,003
PALM2	114299	2,898
ARHN	8153	2,757
MPI	4351	2,637
MGC42105	167359	2,620
GNG2	54331	2,448
YKT6	10652	2,437
BC008967	89927	2,413
RAC3	5881	2,404
GBP2	2634	2,324
OAS1	4938	2,113
GNG7	2788	2,063
GNG10	2790	2,034
LMNB1	4001	1,986
PEX19	5824	1,984
DIRAS1	148252	1,875
RASL10B	91608	1,815
FBXL20	84961	1,755
RHOF	54509	1,752
GNG12	55970	1,700

PPP1R16A	84988	1,615
RAB8A	4218	1,477
GNG3	2785	1,464
C10ORF25	220979	1,429
RHOC	389	1,425
RRAS	6237	1,410
RPGR	6103	1,405
GNG4	2786	1,398
RAB18	22931	1,392
PDE6B	5158	1,375
RAP1B	5908	1,332
CPLX4	339302	1,286
RAC1	5879	1,202
RAB8B	51762	1,152
PTP4A3	11156	1,132
FBXO10	26267	1,119
CAMK1G	57172	0,976
KRAS2	3845	0,957
C17ORF37	84299	0,865
RRAS2	22800	0,755
RALB	5899	0,755
RNF208	727800	0,754
NRAS	4893	0,743
DIRAS2	54769	0,726
GRK7	131890	0,702
ARHI	9077	0,667
RRP22	10633	0,627
GNG8	94235	0,606
CPLX3	594855	0,501
RND1	27289	0,498
FBXL2	25827	0,495
RHEB	6009	0,486
CNP	1267	0,469
RAP2B	5912	0,430
CDC42	998	0,394
DNAJB2	3300	0,287
RALA	5898	0,234
PALM	5064	0,189

Supplementary table 4 | SREBP1 gene signature generated for human samples validation.

ACACB	ACSL4	ACSL5	CSAD
ECHDC1	EHHADH	FADS1	FADS2
GPAM	GSTM1	GSTT1	IGHM
PDK1	RARRES1	SCD	THSRP
